# Intraoperative Fluorescent Navigation of the Ureters, Vessels, and Nerves during Robot-Assisted Sacrocolpopexy

**DOI:** 10.3390/jpm14080827

**Published:** 2024-08-04

**Authors:** Hye Sun Jun, Nara Lee, Bohye Gil, Yoon Jang, Na Kyung Yu, Yong Wook Jung, Bo Seong Yun, Mi Kyoung Kim, Seyeon Won, Seok Ju Seong

**Affiliations:** 1 Department of Obstetrics and Gynecology, CHA Gangnam Medical Center, CHA University School of Medicine, Seoul 06135, Republic of Korea; naradd@chamc.co.kr (N.L.); b223022@chamc.co.kr (B.G.); b223023@chamc.co.kr (Y.J.); b213025@chamc.co.kr (N.K.Y.); sunghunpapa@naver.com (Y.W.J.); ra13811@chamc.co.kr (M.K.K.); drtong85@chamc.co.kr (S.W.); sjseongcheil@naver.com (S.J.S.); 2 Department of Obstetrics and Gynecology, CHA Ilsan Medical Center, CHA University School of Medicine, Goyang 10414, Republic of Korea; bosunyun@chamc.co.kr

**Keywords:** indocyanine green, robot-assisted sacrocolpopexy, apical vaginal prolapse, near-infrared fluorescence imaging, real-time ureter, middle sacral artery, superior hypogastric nerve

## Abstract

In this study, we aimed to demonstrate the feasibility and safety of navigating the ureters, middle sacral artery (MSA), and superior hypogastric nerve (SHN) using indocyanine green (ICG) and near-infrared fluorescence (NIRF) imaging during robot-assisted sacrocolpopexy (RSCP). Overall, 15 patients who underwent RSCP for apical vaginal prolapse were retrospectively enrolled. All patients underwent cystoscopic intraureteric instillation of 5 cc ICG (2.5 mg/mL) before RSCP and intravenous injection of 3 cc ICG during presacral dissection and mesh fixation. In all patients, the fluorescent right ureter was clearly identified in real time. The MSA was visualized on ICG-NIRF images in 80% (13/15) of patients. The mean time from ICG injection to MSA visualization was 43.7 s; the mean duration of the arterial phase was 104.3 s. Fluorescent SHN was detected in 73.3% (11/15) of patients. The time from ICG injection to SHN fluorescence was 48.4 s; the duration of fluorescence was 177.2 s. There was no transfusion, iatrogenic ureteral injury, or bowel or urinary dysfunction. Our results indicated that intraoperative ureter, MSA, and SHN mapping using ICG-NIRF images during RSCP is a valuable and safe technique to avoid iatrogenic ureteral, vascular, and neural injuries and to simplify surgical procedures. Nonetheless, further studies are required.

## 1. Introduction

Pelvic organ prolapse (POP) occurs when one or more structures of the pelvic organs, such as the anterior or posterior vaginal wall or the apex of the vagina, descend into the vaginal space. This condition is commonly referred to as uterine prolapse, cystocele, rectocele, or enterocele. Patients with POP frequently report symptoms such as vaginal bulging, pelvic pressure, dysuria, and defecation disorders, which can significantly affect their quality of life. Although POP can affect premenopausal women, it is most prevalent in women aged 70–79 years, with approximately 13% of women in the US undergoing surgical intervention for POP during their lifetime [1,2,3].

Various surgical strategies, including vaginal and abdominal approaches, have been used to treat POP. Laparoscopic or robot-assisted sacrocolpopexy (RSCP), which uses a mesh rather than native tissue repair, is a well-established and highly effective surgical technique for treating apical vaginal prolapse (AVP). This method is particularly relevant for younger patients with AVP (aged < 60 years), those with advanced AVP beyond pelvic organ prolapse quantification system (POP-Q) stage 3, and obese patients with a high body mass index (BMI), given their high risk of recurrence. However, sacrocolpopexy (SCP) requires a skilled surgeon because of its complexity [4,5].

The first step in SCP is dissection of the presacral area to identify the anterior longitudinal ligament (ALL) and perform promontofixation of the mesh. Critical anatomical structures such as the middle sacral artery (MSA), left common iliac vein, sacral venous plexus (SVP), superior hypogastric nerve (SHN), and right ureter are located around the presacral area, which can lead to complications such as ureteral (1.0%) and vascular injuries (4.4%), resulting in significant blood loss and even death [6,7,8]. Additionally, complications such as bowel, bladder, and sexual dysfunction or pain due to nerve injury can occur [9,10]. Furthermore, there are frequently atypical anatomical variations in the vessels, nerves, and right ureter, including instances of ureteral duplication [11]. Therefore, deep anatomical knowledge and meticulous surgical skills are important in order to avoid life-threatening complications during presacral dissection and mesh suturing [5,12]. Several surgical techniques have been reported for this procedure; these rely heavily on the surgeon’s skills. With recent advancements in minimally invasive surgery, particularly in robotic surgery, young surgeons are increasingly adopting this technique owing to its relatively short learning curve compared to conventional laparoscopy. Nonetheless, robotic surgery has technical challenges because of the intrinsic lack of haptic feedback in the endoscopic approach. Good et al. suggested the use of the sacral promontory, right ureter, and lumbosacral drop as procedural references during promontofixation of RSCP to overcome the difficulties of RSCP [9], but this remains an unresolved concern. Therefore, the simplification of anatomical landmarks and improvement of anatomical structure recognition are essential.

If critical structures such as the right ureter, MSA, and SHN near the presacral space can be visualized in real time using high-signal fluorescent imaging technology during the promontofixation of SCP, presacral dissection and mesh fixation can be performed more safely. This approach would enable even less experienced surgeons to easily perform mesh attachment and reduce intraoperative complications.

Indocyanine green (ICG) is a water-soluble contrast agent approved by the FDA that emits fluorescence in the near-infrared spectrum. It has numerous medical applications, including retinal angiography, organ perfusion assessment, cardiac and hepatic functions quantification, and mapping of the sentinel lymph nodes in gynecologic oncology [13,14,15,16,17,18,19]. Depending on the various surgical sites and the purpose, ICG can be administered through intravenous (IV) injection, peritumoral injection, and urinary tract injection. Following IV administration, ICG attaches to plasma proteins and has a half-life of 3 min, with minimal adverse reactions [13]. Cystoscopic intraureteral (IU) ICG instillation has recently been employed for intraoperative ureteral delineation, thereby reducing the risk of iatrogenic injuries and allowing for 4–12 h of ureteral visualization [20,21,22,23]. Several authors have demonstrated that fluorescent vessel navigation (FVN) using ICG can identify the blood flow in small vessels and minimize unintended arterial injuries during laparoscopic or robotic surgery [24,25]. Kanno et al. highlighted the application of ICG in nerve-sparing techniques during pelvic surgery, emphasizing its potential to improve the outcomes of pelvic endometriosis surgery [26].

In this study, we aimed to demonstrate the feasibility and safety of navigating the right ureter, MSA, and SHN using IU and IV ICG injections along with near-infrared fluorescence (NIRF) imaging during RSCP. This approach prevents iatrogenic ureteral, vascular, and neural injuries; simplifies surgical procedures; and improves patient outcomes.

## 2. Materials and Methods

### 2.1. Patients

This retrospective study enrolled 15 patients who underwent RSCP for apical vaginal prolapse with anterior and posterior compartment prolapse (APCP) between September 2022 and March 2024. All participants had POP-Q stages 2b–4. Patients with a history of ICG or iodide allergy or those with impaired ICG excretion such as liver cirrhosis or use of psychiatric medications were excluded.

Before surgery, all patients underwent a standardized assessment, wherein demographic data and clinical histories were gathered. This included age, BMI, history of medical conditions, parity, menopausal status, past or present menopausal hormonal treatments, previous prolapse or midurethral sling surgeries or hysterectomy, and smoking status. Additionally, a thorough urogynecological evaluation, a physical examination using the POP-Q system, and laboratory tests were performed.

We obtained the following operation-related details: the use of ICG-NIRF imaging mode, concomitant surgery, and the total operation time. Furthermore, we collected data on perioperative outcomes, including estimated blood loss, intraoperative or postoperative complications, the hospitalization period, and any side effects related to IV or IU ICG. Postoperative follow-up data on the POP-Q stage, complications, and toxicity related to ICG were collected until 2 months after surgery.

During RSCP, we monitored the fluorescent ureteral pathway using IU ICG instillation. The MSA, SHN, and SVP were observed using IV ICG-navigated fluorescent imaging during presacral dissection and mesh fixation. The fluorescent images were compared with actual anatomies by reviewing the recorded surgical videos. We also collected data on the initial visualization time and duration of ICG-NIRF images of the right ureter, MSA, and SHN after ICG injection. Following this procedure, short-term outcomes and adverse reactions related to ICG were thoroughly analyzed. All procedures were performed by a single surgeon at the CHA Gangnam Medical Center between September 2022 and March 2024.

ICG (Diagnogreen; Cheil Pharmaceutical Co., Seoul, Republic of Korea) was administered to all patients. We utilized Y-shaped, polypropylene, lightweight mesh (Seratex^®^ PA B2 type; Serag-Wiessner KG, Naila, Germany) according to the study design. The study protocol received approval from the Institutional Review Board of the CHA Gangnam Medical Center (2024-05-007), and the requirement for informed consent was waived owing to the retrospective nature of the study.

### 2.2. Surgical Interventions

All patients underwent standard surgical procedures under general anesthesia. RSCP was performed using the da Vinci Xi system (Intuitive Surgical, Sunnyvale, CA, USA).

Before RSCP, 15 patients with AVP with APCP or vaginal vault prolapse with APCP underwent ICG instillation into the right ureteral orifice using a cystoscopic 6-F open-end catheter. The cystoscopic ICG IU injection steps were similar to those described in our previous report [20]. We used hysteroscopic instruments that are familiar to gynecologists instead of cystoscopes to simplify the preparation without the urologists’ help. Before the surgery, we inserted a hysteroscope (Karl Stortz, Hopkins II ®, Tuttlingen, Germany) into the bladder. A 6-F open-ended ureteral catheter through the hysteroscopic operating channel was placed in the right ureteral orifice. We then instilled 5–6 cc of an ICG mixture (25 mg ICG dissolved in 10 cc of sterile water) into the middle or lower ureter. The catheter was slowly withdrawn. ICG was gradually injected to prevent extravasation. The end of the catheter remained at the ureteral orifice for 1 min to enhance ICG retention and to maximize ICG staining on the ureteral inner lining. In cases in which access to the ureteral orifice was difficult due to pelvic adhesions or ureteral atrophy, guidewire-assisted catheter insertion or IV indigo carmine injection was performed before catheter placement. During robotic surgery, real-time visualization of the fluorescent ureter was performed using the Da Vinci surgical robot.

**Figure 1:**
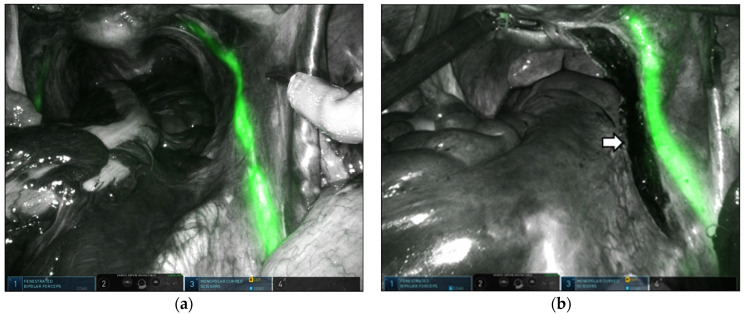
Robot-assisted bilateral ureteral visualization using indocyanine green and near-infrared fluorescence imaging. Right ureter (**a**). Right ureter, with the white arrow showing the retroperitoneal incision from the sacral promontory along the right pelvic side wall to the cul-de sac (**b**).

**Figure 2:**
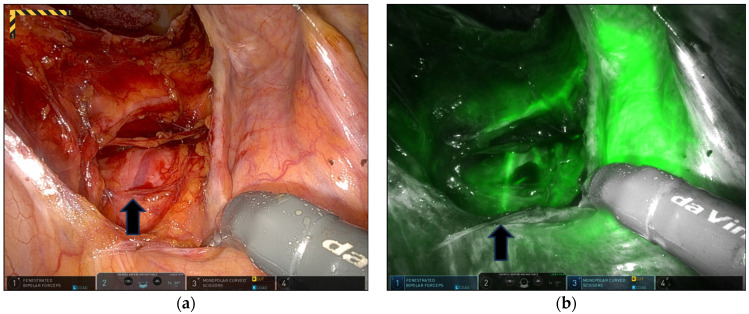
Middle sacral artery on the sacral promontory (indicated by the black arrow). White-light mode (**a**); near-infrared fluorescence imaging using indocyanine green (**b**).

Multiport robotic techniques were executed using three or four ports. The four-port setup comprised an 8 mm umbilical port for the camera, a 10 mm assistant trocar placed subcostally, and two 8 mm ports for the robotic arms positioned on both sides of the umbilicus. The three-port method involved placing a 2 cm glove port (Nelis, Seoul, Republic of Korea) in the umbilicus to accommodate an 8 mm camera port and a 5 mm assistant trocar, along with two 8 mm ports for the robotic arms at the umbilicus level. The robotic patient cart was positioned along the patient’s right side. Sigmoid suspension of the left lateral abdominal wall with V-Loc 2-0 (Covidien, Mansfield, MA, USA) improved the exposure. A retroperitoneal incision was made superficially from the sacral promontory along the right pelvic side wall to the cul-de-sac to create space for burying the mesh. During this procedure, the fluorescent right ureter was highlighted in real time under NIRF imaging to prevent injury to the right ureter (Figure 1). We performed total hysterectomy with or without bilateral salpingo-oophorectomy (BSO). The vaginal vault was closed with barbed sutures using the continuous running suture technique. Thereafter, the vesicovaginal space was dissected to separate the anterior vaginal wall from the bladder. Subsequently, the rectovaginal space was exposed to the perineal body by means of detachment of the posterior vaginal wall from the rectum. We inserted a Y-shaped mesh and secured it to the posterior vaginal wall and the vaginal vault using nine nonabsorbable polypropylene 2-0 sutures (Prolene; Ethicon, Somerville, NJ, USA), a combination of delayed absorbable sutures (2-0 PDS; Ethicon Inc.), or absorbable barbed sutures (2-0 Monofix PDO; Samyang, Daejeon, Republic of Korea, or 2-0 V-Loc; Covidien, Mansfield, MA, USA). The anterior arm of the mesh was then placed on the anterior vaginal wall and sutured with multiple discrete sutures using the same materials as those used on the posterior vaginal wall. Thereafter, 25 mg of ICG was mixed with 10 cc of sterile water, and 3 cc of the ICG solution was injected intravenously. Under fluorescence imaging, the MSA above the midsacral promontory was immediately identified following ICG injection (Figure 2).

**Figure 3:**
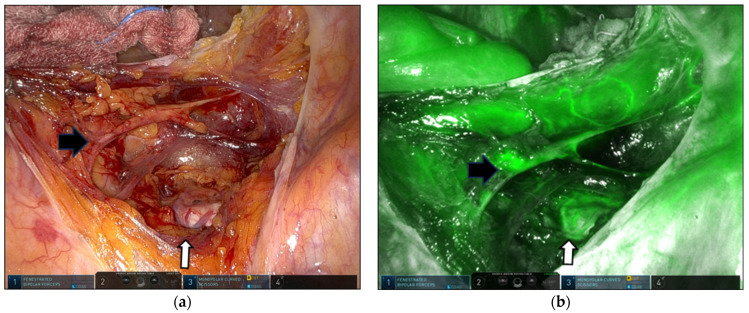
Presacral space. The white arrow indicates the middle sacral artery; the black arrow indicates the superior hypogastric and inferior hypogastric nerves. White-light mode (**a**); near-infrared fluorescence imaging using indocyanine green (**b**).

**Figure 4:**
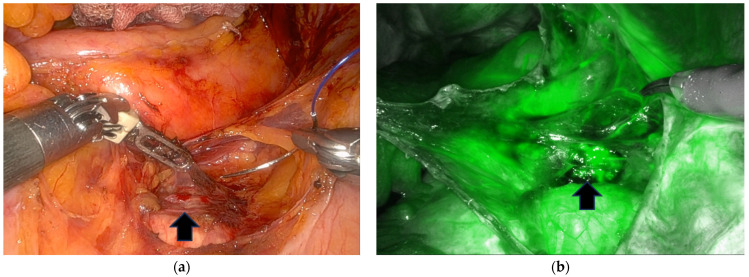
Sacral venous plexus below the sacral promontory (indicated by the black arrow). White-light mode (**a**); near-infrared fluorescence imaging using indocyanine green (**b**).

The superior and inferior hypogastric nerves descending from the left side of the midsacral promontory to the right pararectal space were also visualized (Figure 3). Occasionally, the SVP, located just below the sacral promontory, was detected using ICG fluorescence (Figure 4). While the right ureter, MSA and their vessel variations, and SHN were visualized through ICG-NIRF imaging, we anchored the cranial aspect of the mesh to the ALL at the upper border of the first sacrum below the sacral promontory. This procedure was performed with two to three stitches using nonabsorbable polypropylene 2-0 sutures (Prolene) and nondissolvable polyester 2-0 sutures (Ethibond; Ethicon, Johnson & Johnson, New Brunswick, NJ, USA) after adjusting the mesh tension. The peritoneum was closed using absorbable barbed suture 2-0 (Monofix PDO, Samyang, Daejeon, Republic of Korea) to prevent mesh exposure, bowel adhesion, and bowel strangulation. When clinically indicated, concomitant posterior colpoperineorrhaphy was performed.

All patients were evaluated for (1) the time at which the green ureter was initially visible in fluorescence mode, (2) the extent of visibility downward from the right side of the pelvic brim, and (3) the overall duration of visibility. The differences in clarity were also analyzed. Additionally, all patients were evaluated using video-recorded images to assess (1) the visibility of the MSA, SHN, and SVP following presacral dissection and IV ICG injection; (2) the time interval from ICG injection to first detection; (3) the duration of visibility; and (4) differences in clarity, including the factors that influenced these differences.

After removal of the Foley catheter on the second postoperative day, residual urine was measured, and the patient was discharged after passing stool. Follow-up evaluations were conducted at 2 weeks and 2 months after surgery to assess the POP-Q stage, mesh-related complications, any urinary or bowel dysfunction, and side effects related to ICG.

## 3. Results

### 3.1. Baseline Characteristics

In total, 15 patients were included, consisting of those with POP-Q stage 3 or 4 AVP, young patients under the age of 60 years with POP-Q stage 2b or higher AVP, or AVP patients with intra-abdominal pathology of stage 2b or higher. All AVP patients were associated with APCP. Among them, 14 patients underwent RSCP along with total hysterectomy, and 1 patient underwent robotic BSO with SCP for stage 4 post-hysterectomy vault prolapse. Depending on the correction status after RSCP, three patients underwent additional posterior colpoperineorrhaphy. Adnexectomy was performed in 14 patients, except for 1 patient with previous BSO with myomectomy (Table 1).

The average age of the patients was 63.5 (range, 53–70) years, and the average BMI was 23.5 ± 2.4 (range, 19–27.8) kg/m^2^. One patient had perimenopausal status, and 14 had postmenopausal status. The average number of vaginal deliveries was two (range, one to three), with no cesarean delivery. Three patients (20%) had undergone previous surgery, including laparoscopic BSO and myomectomy (n = 1), total abdominal hysterectomy (n = 1), and urinary stress incontinence surgery using transobturator tape (n = 1). Of 15 patients, 12 had underlying medical illnesses, including hypertension, arrhythmia, chronic obstructive pulmonary disease, and hyperlipidemia. None of the patients had diabetes mellitus or were current smokers. The POP-Q stage was 2b in six patients, 3 in five patients, and 4 in four patients. Nine patients (60%) had pelvic adhesions, and four (26.7%) had adhesions greater than moderate levels. One patient (6.7%) had a severe abdomino-pelvic adhesion. Thirteen patients (86.7%) had concomitant intra-abdominal pathology, including fibroids or adenomyosis in ten and benign ovarian cysts in three patients.

### 3.2. Intraoperative Characteristics

#### 3.2.1. Images of Right Ureter Fluorescence

In all patients, the entire courses of the right or both ureters were visualized in real time during RSCP using IU ICG administration before the surgery. Table 2 shows the results of the ICG fluorescence-navigated right ureter (Figure 1). ICG reversibly stained the inner lining of the ureter by binding to proteins in the urothelial layer [27]. On the ICG-NIRF image, the right ureter was delineated along the right pelvic brim. However, some variations in brightness were noted depending on the depth of the ureter from the peritoneal surface and the patient’s obesity status. ICG-NIRF imaging provided continuous ureteral illumination when the detection of ureteral peristalsis in the white-light mode was challenging because of dense or thick surrounding tissues or dehydration. The fluorescence of the ureters was noticeable immediately after RSCP initiation and generally persisted for 5–8 h. Intraoperative ureteral injuries were not observed in any patient. No intraoperative or postoperative adverse effects on ICG administration were observed during the 2 months of follow-up.

#### 3.2.2. Appearances of MSA and SHN Visualized through ICG Fluorescence

The MSA and SHN were successfully identified during presacral dissection and mesh fixation on ICG fluorescence imaging (Table 2 and Figure 2 and Figure 3).

The peritoneum was opened medially to the right common iliac artery at the promontory level. The peritoneal incision was extended toward the promontory. The underlying presacral fascia and loose connective tissue containing the right SHN and fibers were found and pushed dorsally away from the site of the subsequent incision of the peritoneum. After meticulous dissection, the ALL was exposed, and the MSA and its variations were observed on the ligament. After presacral dissection and ALL exposure, 3 cc of ICG (2.5 mg/mL) was injected intravenously to observe the MSA and its variations. The FVN was compared to the actual anatomy. The time from IV ICG administration to the initial detection of the fluorescent MSA under NIRF imaging and the duration of MSA fluorescence were recorded. As previously reported by Kim et al., the interval time was defined as the time from ICG injection to the initial appearance of the fluorescent artery, and the arterial phase as the time from arterial to venous visualization in the fluorescent mode [25]. The mean interval time was 43.7 (range, 23–89) s, and the average arterial phase lasted 104.3 (range, 14–205) s. The mean total procedural time from ALL identification to suturing the mesh was within 5–10 min. In 12 of the 15 patients (80.0%), ICG-NIRF imaging of the MSA was consistent with the real anatomy (Figure 2 and Figure 3). After IV ICG administration, the ICG-NIRF-navigated SHN nerve was gently displaced to the left during mesh suturing on the ALL to prevent neural impairment (Figure 3). The fluorescent SHN was compared with the actual anatomy. The SHN and its fibers were highlighted by the presence of numerous capillaries surrounding the nerves and fibers. Fluorescent SHNs were identified in 11 of 15 patients (73.3%). The interval from ICG injection to SHN fluorescence was 48.4 (range, 28–89) s, and the duration of fluorescence was 177.2 (range, 49–316) s. In four patients with obesity or in the initial study of this procedure, fluorescent SHNs were not detected (Table 2).

#### 3.2.3. ICG Images of the SVP

Venous visualization was detected at the end of arterial visualization; because the overall fluorescence appeared clumped, the boundary could not be clearly identified except in one or two cases (Figure 4).

### 3.3. Surgical Outcomes

The surgical outcomes are summarized in Table 3. The average operating time was 346 ± 37.9 (range, 260–425) min, and the mean blood loss was 153.3 ± 69.4 (range, 50–300) mL. The decrease in hemoglobin was 1.57 ± 0.80 mg/dL, with a mean preoperative hemoglobin level of 13.0 mg/dL and postoperative hemoglobin level of 11.4 mg/dL. Intraoperative or postoperative blood transfusions were not administered to any patient. Two patients developed mild postoperative pulmonary edema, which improved after 1–2 days. These patients were >70 years old and had underlying medical conditions, including hypertension and arrhythmia. The average length of postoperative hospital stay was 5 (range, 4–7) days. Intraoperative cystotomy, bowel injury, bowel obstruction, wound infection, urinary tract infection, deep vein thrombosis, or hernia was not observed in any patient. No intraoperative or postoperative Clavien–Dindo grade III or higher complications were noted. Additionally, there were no side effects related to ICG for up to 2 months post-surgery. The postoperative POP-Q stage “C” point was −8 to −10. The prolapse of the anterior and posterior compartments associated with AVP was also corrected because of the Y-mesh. No complications associated with the use of this mesh were noted.

## 4. Discussion

In this study, we investigated the feasibility and safety of intraoperative NIRF imaging with ICG to delineate the right ureter, MSA, and SHN in real time during presacral dissection and mesh fixation for RSCP. This is the first study to use intraoperative ICG-NIRF imaging to identify critical structures around the sacral promontory during promontofixation of RSCP in patients with AVP. These findings suggest that these technologies are safe and effective imaging methods for navigating the ureter, MSA, and SHN intraoperatively during promontofixation of RSCP. These interventions may significantly reduce anatomical and functional damage, improve surgical outcomes, and help surgeons make appropriate decisions during complex surgical interventions.

SCP is considered the gold-standard approach for treating advanced AVP; however, it remains one of the most challenging procedures in benign gynecologic surgery and has a long learning curve [28]. The popularity of robot-assisted approaches has complemented the poor ergonomics and extensive learning curve of laparoscopic SCP; however, it has also introduced new challenges due to the lack of tactile feedback. Presacral dissection and mesh attachment are critical steps in SCP, involving important structures such as the ureters, MSA, and SHN. Additionally, the high variability in the location and shape of these structures near the sacral promontory increases the risk of complications, including ureteral injury, significant bleeding, urinary retention, and bowel dysfunction.

Ureteral injury was noted in 1% of SCP cases. The right ureter is located at an average distance of 2.7 cm (range: 1.6–3.8 cm) from the midline of the sacral promontory in cadaver studies and 2.9 cm (range: 1.7–5.0 cm) on computed tomography. Occasionally, the right ureter can be as close as 13 mm from the midsacral promontory [9] and must be identified through ureteral peristalsis during presacral dissection and mesh fixation [12]. However, in obese patients or those with anatomical distortion and severe pelvic adhesions, the course of the ureter may not be visible under the conventional robotic view. Furthermore, electrosurgical energy devices are frequently used in minimally invasive procedures, which can lead to unrecognized ureteral injuries. To prevent intraoperative ureteral injury, surgeons should identify the entire ureter before peritoneal dissection and periodically throughout the surgery [9,12]. Siddighi et al. reported that, through an IU injection of ICG, the ureter’s course could be clearly visualized in >10 patients during RSCP. The authors suggested that this new technique could prevent iatrogenic ureteral damage during pelvic surgery [27]. ICG is a water-soluble tricarbocyanine dye that facilitates the delineation of anatomical structures such as ureters, tumor identification, and lymphatic mapping while also demonstrating promising results in the assessment of organ perfusion and anastomosis. When administered intravenously, ICG is primarily excreted via the liver, bile ducts, and intestines, but not via the kidneys. Typically, ICG has to be administered using a ureteral catheter for intraoperative ureteral identification, and it lasts for 4–12 h [20]. Kim et al. reported that they were able to prevent ureteral injury and minimize blood loss by visualizing the intraoperative fluorescent ureteral pathway in real time through retrograde ICG instillation during complex laparoscopic or robotic gynecologic surgery [20]. We performed ICG-NIRF imaging to identify the right ureter in 15 patients during RSCP with or without hysterectomy. Using ICG-NIRF imaging, the right ureter was clearly visualized in real time along the right pelvic wall in all 15 patients; this visualization persisted throughout the surgery, although ureteral peristalsis was not visible in the white-light mode. Therefore, the right fluorescent green ureter running to the right side of the sacral promontory and the right pelvic sidewall can serve as a new landmark during the longitudinal peritoneal incision from the sacral promontory downward toward the cul-de-sac, especially when the ureter is not visible. This additional cystoscopic procedure typically took 10–20 min with minimal complexity. An extra cost of approximately USD 70 was required for ureteral navigation, including the cost of the ICG drug. However, we believe that the consequent benefits are relatively greater than the additional time and costs.

Hemorrhage is not common, but is a serious complication of SCP, with a reported incidence of 4.4%. These complications typically occur during presacral dissection and suture placement in the ALL [8,29]. The MSA is located 0.2 ± 3.9 mm to the left of the mid-sacral promontory and 48.0 ± 15.4 mm below the aortic bifurcation. This proximity to the mesh attachment site increases the risk of intraoperative damage, which can result in significant bleeding [9,30,31]. To avoid catastrophic hemorrhage during mesh fixation and to enhance the visibility of the MSA, most authors have recommended securing the graft on the ALL at the level of the sacral promontory instead at the S1–S2 or S3–S4 level [9]. Additionally, Good et al. emphasized the need to clearly visualize the needle’s entry and exit points during suturing [9]. Giraudet et al. advocated for an alternative safe zone for mesh fixation at the L5 level to prevent damage to the surrounding structures. The MSA is located close to the midline of the sacral promontory region after incision of the peritoneum and underlying connective tissue; however, the vascular anatomy of the presacral space is extensively aberrant [12]. Surgeons often need to determine promontofixation points based on intraoperative findings. Therefore, if an ICG-NIRF vascular landmark is present in addition to the sacral promontory during presacral dissection and suturing, less experienced surgeons can easily access the landmark and reduce complications related to serious bleeding. Ryu et al. demonstrated that fluorescence-guided vessel navigation is effective for laparoscopic lateral lymph node dissection and can enhance safety by avoiding vessel injury in lower rectal cancer [24]. Using fluorescence imaging, MSA was detected in 12 of the 15 patients (80%). Our findings regarding FVN are consistent with those of Ryu et al. [24]. MSA navigation using ICG-NIRF imaging can provide surgeons with a clearer understanding of the vascular supply in the presacral space, thereby reducing the risk of intraoperative injuries, especially in cases with difficult-to-detect or aberrant vessel anatomy.

Iatrogenic nerve injury is one of the most significant causes of morbidity following surgery. The superior hypogastric nerve and plexus are also located close to the promontory, within 15–30 mm [7]. During laparoscopic surgery or RSCP, dissection of the presacral space can damage the SHN and fibers, potentially resulting in postoperative events such as micturition disorders, pelvic pain, defecation problems, and sensory disturbances [32]. The SHN is located immediately under the peritoneum, to the left of the midline. Sarlos et al. identified and preserved the SHN during presacral dissection of laparoscopic SCP. The critical step involves visualizing and carefully moving the SHN and fibers to the left at the promontory level while attaching the mesh to the ALL [32]. The relatively thin hypogastric nerve is surrounded by connective tissues just beneath the peritoneum, making it difficult for even experienced surgeons to detect it. Although ICG is not a nerve-specific dye, Kanno et al. reported that it was useful for nerve-sparing procedures during laparoscopic endometriosis surgery [26]. It can easily identify ischemic endometriotic nodules and blood-supplied nerves. The neurovascular bundles comprise interconnected nerve terminals and capillaries. Intravenous ICG injection can be used to detect capillaries in the neurovascular bundle and indirectly assess nerves by observing blood flow [33]. The nerve was assessed using ICG uptake to objectively evaluate perfusion, ensuring that the superior and inferior hypogastric plexuses were mapped in green by ICG, allowing nerve-sparing surgery. We observed fluorescent SHN in 11 (73.3%) of 15 patients. The high variability in the locations of anatomical structures, such as vascular or neural elements, and the thickness of the overlying tissues may have resulted in a relatively lower detection rate compared with that of the ureter. The delineation of the ureteral pathway using IU ICG instillation was maintained for a sufficient duration in real time without a change in clarity during surgery, aided by additional cystoscopic procedures. In contrast, the IV administration of ICG is simple but has a short fluorescence duration. When the arterial phase transitions to the venous phase, surrounding tissues become progressively fluorescent, leading to the loss of distinct vessel and nerve boundaries [25]. To identify MSA and SHN, ICG should be administered immediately before mesh suturing under NIRF imaging. Despite the short fluorescence durations for the MSA and SHN, navigating their locations before mesh suturing can effectively prevent nerve entrapment and bleeding complications. The time required for mesh suturing was less than 5 min in our study. This should be considered when developing future fluorophores for vessel or nerve navigation to overcome the limitations of the rapid transit time of ICG. Further studies of patient factors, fluorophore properties, administration routes and volumes, and fluorescence intensity are required. Although our techniques require further research, they can help to identify and protect the ureters, MSA, and SHN during SCP.

The advantages of this study are summarized as follows. This is the first study to use intraoperative NIRF imaging with ICG to identify the ureters, MSA, and SHN during promontofixation of RSCP in AVP. This technique can reduce the learning curve by guiding the identification of critical structures around the sacral promontory using fluorescent images, thereby simplifying complex, hazardous, and time-consuming procedures and making them more accessible to novice surgeons. Second, because this study was conducted by a single surgeon from diagnosis to surgery to postoperative monitoring, any inter-individual discrepancies arising from differences in surgical expertise were minimized.

This study has several limitations. First, we did not conduct a randomized controlled trial to compare the identification and injury rates of the ureter, vessels, and nerves between NIRF-navigated surgeries and traditional methods. Second, the analysis was restricted to a limited number of cases, which affected the generalizability of the results. Consequently, the level of evidence should be enhanced by extending its application to larger cohorts across multiple centers. In the future, this imaging platform should be applied to nerve-sparing endometriosis surgery, gynecologic oncology, and blood-flow evaluation following uterine transplantation [34,35]. Furthermore, studies should evaluate patient factors affecting the noise-to-background ratio, including diabetes, smoking status, and obesity, as well as the fluorescence intensity based on ICG dosage and administration method [36]. Despite being a small-scale study with short-term outcomes, this study may provide sufficient insight into the effectiveness and safety of ICG-NIRF navigation for identifying ureters, vessels, and nerves during SCP.

## 5. Conclusions

Fluorescence visualization of the right ureter in real time during RSCP was made possible using IU ICG instillation. Intraoperative fluorescence navigation of the MSA and SHN, along with their variations, was briefly visible after IV ICG administration during mesh promontofixation. This technology provides valuable intraoperative landmarks for both highly skilled and novice surgeons to avoid ureteral, vascular, and neural injuries during presacral dissection and mesh fixation. Further studies are required in order to validate the potential application of intraoperative ICG-NIRF imaging during RSCP.

## Figures and Tables

**Table 1:** Baseline characteristics (sociodemographic data and clinical history) of the patients.

Variable	n = 15
Age, years, median, and range	63.5 (53–70)
Vaginal parity, median, and range	2 (1–3)
BMI, kg/m^2^, mean ± SD	23.5 ± 2.4
Menopause or perimenopause, n (%)	15 (100%)
Associated medical illness, n (%)	
Hypertension	12 (80%)
Arrythmia	2 (13.3%)
Angina	2 (13.3%)
Early COPD	1 (6.7%)
Hyperlipidemia	5 (33.3%)
Fatty liver	3 (20%)
Previous pelvic surgery, n (%)	
Total abdominal hysterectomy	1 (6.7%)
Salpingo-oophorectomy and myomectomy	1 (6.7%)
Previous midurethral sling	1 (6.7%)
Concomitant intra-abdominal pathology	13 (86.7%)
Fibroids or adenomyosis	10 (66.7%)
Benign ovarian cysts	3 (20%)
POP-Q at baseline ≥ IIb	
Anterior	15 (100%)
Apical	15 (100%)
Posterior	3 (20%)
POP-Q staging, n (%)	
Stage IIb	6 (40%)
Stage III	5 (33.3%)
Stage IV	4 (26.7%)

BMI: body mass index, SD: standard deviation, COPD: chronic obstructive pulmonary disease, POP-Q: pelvic organ prolapse quantification (POP-Q).

**Table 2:** Results of the ICG fluorescence-navigated ureter, MSA, and SHN.

Variable	n = 15
Visualization of the ureter	15/15 (100%)
Time from ICG injection to fluorescence	Immediately
Duration of fluorescence (h)	>5 h
Visualization of the MSA	12/15 (80%)
Time from ICG injection to fluorescence (s)	43.7 (23–89)
Duration of MSA fluorescence (s)	104.3 (14–205)
Visualization of the SHN	11/15 (73.3%)
Time from ICG injection to fluorescence (s)	48.4 (28–89)
Duration of SHN fluorescence (s)	177.2 (49–316)

Values are presented as numbers (%) or medians (ranges). MSA: middle sacral vessel, SHN: superior hypogastric nerve, ICG: indocyanine green.

**Table 3:** Operative outcomes.

Variables	n = 15
Total operation time ^1^, (minutes), mean ± SD	346 ± 37.9
Concomitant surgery, n (%)	15 (100%)
Total hysterectomy	14 (93.3%)
Adnexectomy	14 (93.3%)
Adhesiolysis	9 (60%)
Posterior colpoperineorrhaphy	3 (20%)
Estimated blood loss (mL), mean ± SD	153.3 ± 69.4
Hemoglobin decrease (g/dL), mean ± SD	1.57
Transfusion	0
Hospital length of stay (days), median and range	5 (4–7)
Clavien–Dindo grade III or higher complications	0

^1^ Total operation time was defined as the time interval from skin incision to the completion of skin closure, including all concomitant operations, SD: standard deviation.

## Data Availability

Data are available upon reasonable request from the corresponding authors. However, the data cannot be made public to protect the women’s privacy for legal reasons because they contain private health information along with identifiers.
